# Monoclonal Antibodies against SARS-CoV-2: Current Scenario and Future Perspectives

**DOI:** 10.3390/ph14121272

**Published:** 2021-12-06

**Authors:** Eugenia Quiros-Roldan, Silvia Amadasi, Isabella Zanella, Melania Degli Antoni, Samuele Storti, Giorgio Tiecco, Francesco Castelli

**Affiliations:** 1 Department of Infectious and Tropical Diseases, University of Brescia and ASST Spedali Civili di Brescia, Piazzale Spedali Civili 1, 25123 Brescia, Italy; silvia.amadasi@live.it (S.A.); m.degliantoni@unibs.it (M.D.A.); s.storti@unibs.it (S.S.); g.tiecco@unibs.it (G.T.); francesco.castelli@unibs.it (F.C.); 2 Clinical Chemistry Laboratory, Diagnostic Department, Department of Molecular and Translational Medicine, University of Brescia and ASST Spedali Civili di Brescia, Piazzale Spedali Civili 1, 25123 Brescia, Italy; isabella.zanella@unibs.it

**Keywords:** SARS-CoV-2, monoclonal antibodies, variants, prophylaxis, therapy

## Abstract

Monoclonal antibodies (mAbs) have been known since the 1970s. However, their therapeutic potential in the medical field has recently emerged, with the advancement of manufacturing techniques. Initially exploited mainly in the oncology field, mAbs have become increasingly relevant in Infectious Diseases. Numerous mAbs have been developed against SARS-CoV 2 and have proven their effectiveness, especially in the management of the mild-to-moderate disease. In this review, we describe the monoclonal antibodies currently authorized for the treatment of the coronavirus disease 19 (COVID-19) and offer an insight into the clinical trials that led to their approval. We discuss the mechanisms of action and methods of administration as well as the prophylactic and therapeutic labelled indications (both in outpatient and hospital settings). Furthermore, we address the critical issues regarding mAbs, focusing on their effectiveness against the variants of concern (VoC) and their role now that a large part of the population has been vaccinated. The purpose is to offer the clinician an up-to-date overview of a therapeutic tool that could prove decisive in treating patients at high risk of progression to severe disease.

## 1. Introduction

Since 1901, when Emil Adolf von Behring won the Nobel Prize in Medicine for the application of animal-derived serum therapies, this approach has been attempted for several emerging infectious diseases, based on the pivotal role of the serological immune response against infectious diseases [1]. Antibodies are now a versatile tool for diagnostics and therapy of various conditions in humans and hyperimmune sera can be replaced by specific monoclonal antibodies (mAbs). MAbs were first described almost half a century ago, deriving from mice’s vaccination with specific antigens followed by B cells harvesting from mouse spleens. Now, antibodies have become significantly easier to develop and produce whereby relevant antibodies can be identified directly from exposed persons. Investigators are now able to use flow cytometry to distinguish memory B cells based on their antigen-binding characteristics [2]. The variable regions of the antibody heavy and light chains can be replicated, achieving monoclonal antibodies expression [3]. From these initial procedures, candidates for mAbs development can be further selected according to in vitro neutralization assays that assess their activity. Thanks to the above mentioned progress in techniques, the necessary time to isolate and characterize antibodies has been significantly reduced.

A mAb with potential therapeutic utility should fulfill the following three conditions at least: (i) the antigen-binding fragment (Fab) domain must specifically bind to the appropriate molecular target, (ii) efficient and precise effector functions activated by binding of the constant crystallizable fragment (Fc) region to specific receptors of immune cells, and (iii) good pharmacokinetic characteristics [1].

Initially, the application of mAbs was restricted to the development of diagnostic techniques with limited therapeutic applications, because mAbs were of animal origin exclusively, burdened by potential downsides in terms of high immunogenicity, limited half-life, and scarce capacity of activating Fc-mediated effector functions after administration. These problems were addressed by the engineering of the constant regions of an antibody molecule leading first to chimeric (murine mAbs with a human Fc fragment) and then to humanized antibodies (human mAbs, maintaining the complementarity determining regions of the original mouse mAbs) [4]. In the last two decades, novel techniques introduced the possibility of dissecting directly the human “antibodyome,” allowing the selection of fully human mAbs [4].

Advances in mAbs research and production could have an enormous impact in the field of medicine. Although initially devised for infectious diseases prevention or treatment, mAbs are now more extensively applied in other clinical areas as oncology or immunology.

Theoretically, mAbs could be employed against a broad variety of biologic agents, encompassing bacterial and viral pathogens, fungi, and associated toxins, with their action exerted either directly (e.g., preventing cell entry or neutralizing toxins) or via indirect mechanisms (e.g., modulating inflammatory responses or promoting opsonic phagocytosis) [5].

Palivizumab was the first mAb approved in Europe in 1999, licensed for prophylaxis against the serious respiratory disease caused by respiratory syncytial virus (RSV) in infants at high risk for severe manifestations. Palivizumab, which recognizes an epitope in the fusion protein was shown to reduce hospitalization by 55% in both premature infants and those with bronchopulmonary dysplasia [6,7]. Only twenty years later, bezlotoxumab (which targets *Clostridioides difficile* toxin B), the second mAb in the field of infectious diseases, was authorized in Europe to prevent recurrences of infection in adults at high risk of repeated bouts [8].

Despite their potentially extensive impact, up to date, only few mAbs are licensed against infectious agents or toxins. This important field of research is constantly expanding, and new drugs are being licensed for therapeutic applications in the area of infectious diseases. For example, the FDA approved raxibacumab and obiltoxaximab, for the treatment and prophylaxis of inhalational anthrax, respectively, when alternative therapies are not available or not appropriate [9,10]. Moreover, ibalizumab was recently licensed as rescue therapy in heavily treatment-experienced adults with multidrug-resistant HIV-1 infection [11].

During the Ebola virus outbreak in West Africa in 2014–2016, several mAbs were investigated as a possible weapon against the virus. Several mAbs cocktails were developed, including ZMapp (a combination of three chimeric mAbs produced in the plant Nicotiana benthamiana), REGN-EB3 (a combination of three mAbs produced in humanized mice that bind to non-overlapping epitopes of Ebola virus glycoprotein), and a single monoclonal antibody (mAb114, which targets the receptor-binding domain of the virus glycoprotein). Efficacy and tolerability were tested in the Pamoja Tulinde Maisha (PALM) trial: a randomized, controlled trial, initiated in February 2015. However, as the incidence of Ebola infection dropped, trial enrollment decreased, and the study failed to reach statistical significance. Although limited, the data showed safeness and the possibility of a mortality benefit. The interim results of the trial suggested a significant improvement of survival for patients receiving mAb114 (64% survival rate) or REGN-EB3 (66.5% survival rate), compared to those receiving ZMapp (49.5% survival rate) [12]. Notably, patients who received early care and treatment fared better than those who were treated later.

## 2. Antibodies Targeting SARS-CoV-2

The severe acute respiratory syndrome coronavirus 2 (SARS-CoV-2) continues to spread worldwide as a severe pandemic [13]. The development of therapeutic mAbs is currently at the front line of fighting against the coronavirus disease 19 (COVID-19) pandemic and hundreds of therapeutic antibodies are either in the preclinical stages or in clinical trials [14]. Owing to the close relatedness of SARS-CoV-2 to SARS-CoV-1, initial efforts of mAbs therapy against the former focused on repurposing anti-SARS-CoV-1 mAbs with cross-neutralizing activity against SARS-CoV-2. Later, memory B cells specific to the receptor-binding-domain (RBD) of SARS-CoV-2 spike protein were used to generate SARS-CoV-2 specific IgG1 mAbs that exhibit potent neutralizing activity [15,16,17,18].

**Figure 1:**
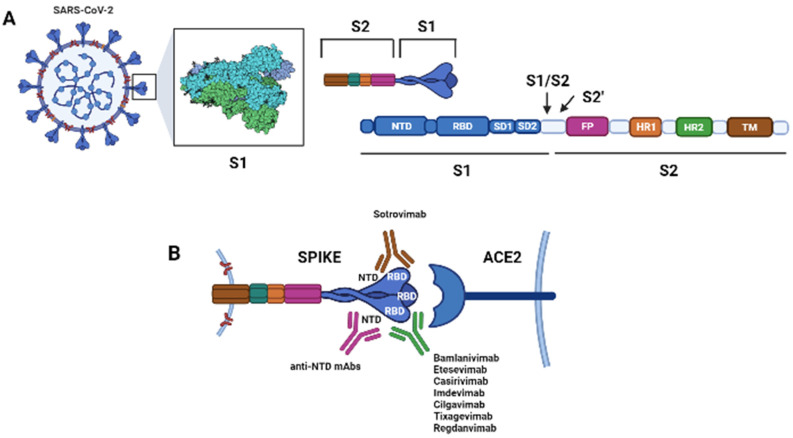
Schematic of the spike protein (S) of Sars-CoV-2 and its interactions with its cellular receptor, the angiotensin converting enzyme 2 (ACE2), and with therapeutic monoclonal antibodies (mAbs). The S protein is a trimer. (**A**) Each monomer of the S protein consists of a N-terminal S1 subunit [comprising the N-terminal domain (NTD), the receptor-binding domain (RBD), subdomain 1 (SD1), and subdomain 2 (SD2)] and a C-terminal S2 subunit [comprising the fusion peptide (FP), the heptad repeat 1 (HR1), the heptad repeat 2 (HR2) and the transmembrane domain (TM)]. The S1 subunit binds the ACE2 receptor, while the S2 subunit is involved in membrane fusion during cell entry. Upon binding of the trimer to the host cell receptor through the RBD, the S1 and S2 subunits are cleaved by the host transmembrane protease serine 2 (TMPRSS2) at the S1/S2 junction; then, a second site within the S2 subunit, termed the S2′ site, is cleaved by serine proteases or cathepsins and viral-host membranes fusion is initiated. (**B**) Interaction between the S protein and the host cell receptor ACE2. Most therapeutic mAb targets the RBD of the S protein at positions required for the interaction with ACE2 (Bamlanivimab, Etesevimab, Casirivimab, Imdevimab, Cilgavimab, Tixagevimab, Regdanvimab) while Sotrovimab targets the RBD, but does not compete with human ACE2 receptor binding. mAbs binding the NTD have been demonstrated to neutralize SARS-CoV-2, and these could be developed for therapeutic purposes [15,19,20,21,22,23,24,25,26] (Created with BioRender.com).

The spike (S) protein on the surface of SARS-CoV-2 binds the host cellular angiotensin-converting enzyme 2 (ACE2) receptor, allowing the virus to infect host cells. The S protein is a trimer, and each monomer comprises an N-terminal S1 subunit and a C-terminal S2 subunit. The S1 subunit further separates into the N-terminal domain (NTD), subdomain 1 (SD1), subdomain 2 (SD2) and RBD. The S2 subunit further divides into the fusion peptide (FP), the heptad repeats 1 (HR1), and heptad repeat 2 (HR2) [Figure 1]. During the virus entry phase, S1 is responsible for the receptor binding, while S2 is involved in the membrane fusion.

The S protein can be in a receptor inaccessible (closed, pre-fusion) or accessible (open, post-fusion) state according to the position of the RDB. In its open conformation, the RBD domain is exposed and able to bind ACE2; in the closed conformation, it is instead blocked.

Most neutralizing antibodies target S1, particularly the S1-RBD, due to its important function as well as its significant immunogenicity [27,28].

Additionally, antibodies binding the N-terminal domain of the S-protein, or a distinct proteoglycan epitope have been demonstrated to neutralize SARS-CoV-2, and could be developed for therapeutic purposes [15,19,20,21,22,23,24,25,26]. Two mAbs developed against MERS-CoV, G2 and 7D10, target the S1-NTD region blocking the interaction between the spike protein and the host receptor DPP4. The coronavirus S2 subunit is more conserved than the S1, and carries epitopes that may represent a potential target for broadly neutralizing antibodies [Figure 1].

The antiviral effect of mAbs is the outcome of their two functional domains: Fab that confers antigen specificity, responsible for virus neutralization, and the Fc that drives effector functions. The majority of mAbs with activity against SARS-CoV-2 bind to the S1 subunit and inhibit the virus engagement to its cell surface receptor, ACE2, with their Fab domain (neutralizing activity).

The Fc domain (constant domain) engages complement or Fc gamma receptors (FcγRs) on leukocytes promoting immune-mediated cellular clearance, the enhancement of antigen presentation and CD8+ T cell responses. Fc-FcγR interactions proved to be essential for the in vivo antiviral activity of anti-SARS-CoV-2 mAbs. MAbs lacking Fc domain showed reduced antiviral activity in vivo [29].

Additionally, the Fc domain can reshape inflammation through engagement of FcγRs on specific cells [30]. In particular conditions, Fc-FcγR interactions may cause antibody-dependent enhancement (ADE) of virus infection or pathological immune skewing. The effect of ADE mediated by the Fc receptor (FCR) mainly involves the interaction between the Fc fragment of the antibody and the FcγR on the cell surface. This allows the virus and antibody complex to combine with cells that express FcγR, causing the virus to adhere to their surface and increase the risk of infection.

Current evidences do not support the hypothesis that SARS-CoV-2 infection, mAbs against SARS-CoV-2 or vaccination may cause ADE effects. Although unlikely, the possibility of ADE compels a careful consideration on the role of the antibody Fc in SARS-CoV-2 therapeutic design [31].

The neonatal Fc receptor (FcRn) is an MHC class I like molecule associated with beta-2-microglobulin (β2m) and it is involved in the IgG turnover regulating the antibody half-life in vivo mediating bidirectional transcytosis of IgG across epithelial cells as well as membrane recycling. Fc engineering has exploited the FcRn-mediated IgG recycling to develop mAbs therapeutics with improved pharmacokinetic properties, allowing lowered dosing by extending half-life and increased potency [32].

### 2.1. Principal mAbs against SARS-CoV-2

Several mAbs with neutralizing activity against SARS-CoV-2 have showed clinical benefit in cases of mild to moderate COVID-19, reducing the risk of hospitalization and severe disease. Nonetheless, their effectiveness in preventing disease complications or mortality among COVID-19 hospitalized patients was limited. Hereafter, we briefly describe SARS-CoV-2 RBD-specific neutralizing antibodies commercialized for clinical use.

#### 2.1.1. Bamlanivimab

Bamlanivimab (also known as LY-CoV555 and LY3819253) is a recombinant neutralizing human IgG1k mAb, unmodified in the Fc region and produced by Eli Lilly. It targets the receptor binding domain of the S-protein of SARS-CoV-2. It was originally derived from the blood of one of the first U.S. patients who recovered from COVID-19. X-ray crystallography and cryo-EM structural determination suggest that bamlanivimab binds the RBD of the S protein at a position overlapping the ACE2 binding site, which is accessible in both the up and down conformations of the RBD.

Bamlanivimab binds the spike protein with a dissociation constant K_D_ = 0.071 nM and blocks the S protein attachment to the human ACE2 receptor with an IC_50_ value of 0.025 µg/mL. The authorized dosage is a single intravenous infusion of 700 mg administered within 10 days of symptoms onset [33].

In April 2021, the Food and Drug Administration (FDA) revoked the Emergency Use Authorizations (EUA) for bamlanivimab alone, due to the increasing circulation of SARS-CoV-2 variants [34].

#### 2.1.2. Bamlanivimab and Etesevimab

Etesevimab (also known as LY-CoV016 and LY3832479 and JS016) is also an mAb manufactured by Eli Lilly that binds to a different but overlapping epitope in the RBD of the SARS-CoV-2 S protein. Etesevimab is a recombinant neutralizing human IgG1κ mAb to the S protein of SARS-CoV-2, with amino acid substitutions in the Fc region (L234A, L235A) to reduce effector function. Etesevimab binds the S protein with a dissociation constant KD = 6.45 nM and blocks S protein attachment to the human ACE2 receptor with an IC50 value of 0.32 nM (0.046 µg/mL) [35]. Bamlanivimab is able to bind RBD in both its open and closed conformations, while etesevimab binds only to the open conformation. Because of RBD fluctuation between its two configurations, targeting both allows for greater neutralization.

Therefore, its use was initially approved in combination with bamlanivimab (dosage of bamlanivimab/ etesevimab: 700/1400 mg), because employing both antibodies together was expected to reduce the risk of viral resistance.

The authorized treatment dosage is 700 mg of bamlanvimab and 1400 mg of etesevimab administered together as a single intravenous infusion [36].

#### 2.1.3. Casirivimab and Imdevimab

Casirivimab (previously REGN10933) and imdevimab (previously REGN10987) are manufactured by Regeneron [37].

Casirivimab and imdevimab are two human immunoglobulin G-1 (IgG1) mAbs, unmodified in the Fc regions. They bind distinct epitopes of the spike protein RBD of SARS-CoV-2 with dissociation constants KD = 45.8 pM and 46.7 pM, respectively. They block RBD attachment to the ACE2 receptor with IC50 values of 56.4 pM, 165 pM, respectively, and prevent viral attachment to host cells.

The authorized treatment dosage is 600 mg of casirivimab and 600 mg of imdevimab administered together as a single intravenous infusion. Moreover, if the intravenous infusion is not achievable and would cause a treatment delay, subcutaneous injection is authorized as an alternative route of administration [38].

#### 2.1.4. Sotrovimab

Sotrovimab (previously VIR-7831 and also known as GSK4182136 and S309) is the last mAb approved by FDA, produced by GlaxoSmithKline. It was originally identified in 2003 from a SARS-CoV survivor. It targets an epitope in the RBD of the S protein that is conserved between SARS-CoV and SARS-CoV-2. It is a recombinant human IgG1-kappa mAb that binds to a conserved epitope on the S protein RBD of SARS-CoV-2 (dissociation constant K_D_ = 0.21 nM), but does not compete with human ACE2 receptor binding (IC_50_ value > 33.6 nM [5 µg/mL]). Sotrovimab inhibits an as yet undefined step that takes place after the virus attachment and before the fusion of the viral and cell membranes. The Fc domain of sotrovimab includes two amino acid substitutions (LS modification), M428L and N434S that prolong antibody half-life, but do not affect wild-type Fc-mediated effector functions in cell culture.

The authorized dosage for sotrovimab is one single intravenous infusion of 500 mg [39].

#### 2.1.5. Cilgavimab and Tixagevimab

This is a long-acting antibody combination (AZD7442) manufactured by Astrazeneca, constituted by cilgavimab (COV2-2130 or AZD1061) and tixagevimab (COV2-2196 or AZD8895). These two non-competing antibodies synergistically neutralized SARS-CoV-2 in vitro and protected against SARS-CoV-2 infection in mouse models and a rhesus macaque model when used separately or in combination [40,41]. Several phase 3 clinical trials are ongoing to study this association for post-exposure prophylaxis (NCT04625972), prevention (NCT04625725), outpatient (NCT04723394 and NCT04518410) and inpatient (NCT04501978) treatment of COVID-19 [42].

This mAb cocktail is currently being tested in an early-therapy study and in a large pre-exposure prophylactic study enrolling up to 5000 participants administered with a low dose of the AZD8895/AZD1061 cocktail (0.15 + 0.15 g of each mAb) that is compatible with intramuscular delivery. This trial will evaluate the incidence of SARS-CoV-2 PCR-positive symptomatic illness against placebo for a period up to 6 months (trial number NCT04625725). The results will be crucial to assess the optimal use of this combination in prophylactic or therapeutic settings. The use of low doses is essential to allow the subcutaneous or intramuscular administration of low volumes of high concentration product.

In October 2021, Astrazeneca submitted a request to US FDA for EUA for AZD7442 for prophylaxis for COVID-19.

#### 2.1.6. Regdanvimab

Regdanvimab (CT-P59) is a mAb produced by Celltrion Healthcare Hungari Kft. It blocks interaction regions of RBD for ACE2 with an orientation that is notably different from previously reported RBD-targeting mAbs. The therapeutic effects of CT-P59 were assessed in animal models (ferret, hamster, and rhesus monkey), showing a considerable decrease in viral titer as well as an improvement in clinical symptoms. Therefore, CTP59 may be a promising therapeutic candidate for COVID-19 [43].

In September 2021, the South Korea Ministry of Food and Drug Safety (MFDS) has approved regdanvimab to treat mild COVID-19 in patients aged 50 years and above with a minimum of one underlying medical condition, and moderate symptoms of the disease in adults. This approval was based on the first part of a global phase 2/3 trial showing that progression rates to severe COVID-19 were reduced by 54% for patients with mild-to-moderate symptoms and 68% for patients aged 50 years and older [44].

In October 2021, the European Medical Agency (EMA) has started considering an application for marketing authorization for this mAb to treat adults with COVID-19 who do not require supplemental oxygen therapy and who are at increased risk of progressing to severe COVID-19.

The dosage for regdanvimab is one single intravenous infusion of 40 mg/kg [45].

The phase 2 BLAZE-4 trial (NCT04634409) a double-blind, placebo-controlled randomized trial is assessing the efficacy and safety of bamlanivimab (700 mg) with others mAbs including sotrovimab (500 mg) for treatment of symptomatic low-risk COVID-19 non-hospitalized patients. Preliminary results showed that bamlanivimab/sotrovimab (700/500 mg) demonstrated a 70% (*p* < 0.001) relative reduction in persistently high viral load (>5.27; cycle threshold value <27.5 at day 7 compared to placebo) [46].

A recent study compared and rated according to “confidence in the evidence” all published studies that assessed SARS-CoV-2-neutralising mAbs (alone or combined, compared to an active comparator, placebo, or no intervention) to treat people with COVID-19 (excluding studies on prophylactic use). The authors concluded that the available evidence is insufficient to draw meaningful conclusions regarding treatment with SARS-CoV-2-neutralising mAbs [14].

### 2.2. MAbs Approved against SARS-CoV-2 by Emergency Use Authorizations (EUAs)

EUA of mAbs against SARS-COV-2 were due to the context declared emergency without available alternatives. EUA is a mechanism used by the FDA to facilitate making products available quickly during a public health emergency; this differs from FDA Approval, which is an independent, scientifically reviewed approval for medical products, drugs, and vaccines, based on substantial clinical data and evidence.

Three regimens currently have EUA from the FDA for the treatment of mild to moderate COVID-19 in non-hospitalized adults and pediatric patients (12 years of age and older weighing at least 40 kg) with laboratory-confirmed SARS-CoV-2 infection who are at high risk for progression to severe disease and hospitalization. Risk factors for progression to severe COVID-19 are reported in Table 1. The use of SARS-CoV 2 neutralising antibodies has not been authorized by the FDA-EUAs for patient hospitalized for COVID-19 or for those requiring oxygen therapy due to COVID-19 or patient who are on chronic oxygen therapy due to an underlying condition not related to COVID-19 that require an increase in oxygen flow rate from baseline. Furthermore, the FDA EUAs indicates that all approved mAbs may be associated with worse clinical outcomes when administered to hospitalized patients with COVID-19 requiring high flow oxygen or mechanical ventilation [34,36,38,39].

The products currently approved with EUA are:

- Bamlanivimab 700 mg plus etesevimab 1400 mg (intravenous infusion, (iv));
- Casirivimab 600 mg plus imdevimab 600 mg (iv or subcutaneous injection);
- Sotrovimab 500 mg (iv).

The main randomized clinical trials supporting FDA EUAs are summarized in Table 2.

Phase 3 of BLAZE-1 trial (NCT04427501) is a double-blind, placebo-controlled randomized trial. The study involved outpatients with mild to moderate COVID-19 who were at high risk for progressing to severe COVID-19 and compared a single IV infusion of bamlanivimab 2800 mg plus etesevimab 2800 mg versus placebo, administered within 3 days of a positive SARS-CoV-2 virologic test. In the bamlanivimab plus etesevimab arm, the trial showed a 4.8% absolute reduction and a 70% relative reduction in hospitalizations due to COVID-19 or deaths from any cause [48]. The authorized dosage of 700/1400 mg lower than the dosage tested in BLAZE-1 is based on initial results from the ongoing BLAZE-4 trial (NCT04634409) which demonstrate that bamlanivimab/etesevimab dosage of 700/1400 mg provides viral load and pharmacodynamic/pharmacokinetic data similar to the dose of 2800/2800 tested in BLAZE-1 trial [46].

The EUA of intravenous casirivimab 600 mg plus imdevimab 600 mg is based on the Phase 3 results from the Regeneron-COV-2067 trial (NCT04425629). This study is a double-blind, placebo-controlled randomized trial in outpatients with mild to moderate COVID-19 at risk for progression to severe COVID-19. An absolute reduction of 2.2% and a 70% relative reduction in hospitalization or death was demonstrated among the participants that received mAbs compared to those who received placebo. These findings are similar to those observed for IV infusions of casirivimab 1200 mg plus imdevimab 1200 mg (3.3% absolute reduction and a 71% relative reduction in hospitalization or death compared to the placebo arm) [49,50].

Sotrovimab is supported by the results coming from interim analysis of the ongoing, multicenter, double-blind, phase 3 COMET-ICE trial (NCT04545060); 583 participants at high risk for progression to severe disease were randomized to receive sotrovimab 500 mg IV or placebo. The trial showed an 85% relative risk reduction in hospitalization or death among those receiving sotrovimab instead of placebo. Moreover, serious adverse events were fewer in the sotrovimab arm compared to the placebo arm [51].

The main limitation of these trials is their heterogenicity in settings and reported outcomes that renders difficult the establishment of a proper comparison. Additionally, important information on mAbs adverse effects and reports on the quality of life of the treated patients are still lacking [14].

## 3. Therapeutic and Prophylactic Indications

### 3.1. Prophylactic Use of mAb against SARS-CoV-2

Vaccines undeniably represent the most effective way to provide protection from COVID-19 for most individuals. For the last two years, the whole scientific community focused on the research, development and, ultimately, production, of a safe and effective vaccine, because it represented the only possible solution to further spreading and recurrence of SARS-CoV 2. [52]. Vaccine development can take years, or even decades, but the aggressive efforts made to investigate several COVID-19 vaccine candidates concomitantly may significantly reduce the amount of time generally required for the development process [53]. MAbs currently represent an alternative route of prevention for COVID-19 and could offer short-term protection to those who are not yet vaccinated or who lack a proper response to vaccination, such as immunocompromised patients. Additionally, mAbs could prove helpful during times when circulating variant viruses are not adequately covered by vaccines protection [54].

Moreover, because a certain time is required after vaccination to develop a proper immune response, the benefits of passive immunization are evident in numerous settings where outbreaks are frequent, such as health care facilities and households [55].

Several ongoing prophylaxis studies evaluated the potential role of mAbs for prevention of infection or symptomatic disease, with interesting data from nursing homes and households that demonstrated their beneficial role in post-exposure prophylaxis.

The phase 3 BLAZE-2 trial (NCT04497907) was a randomized, double-blind, placebo-controlled trial designed to evaluate the efficacy and safety of bamlanivimab (4200 mg, iv) alone or in combination with other mAbs in preventing COVID-19 in skilled nursing and assisted living facility residents and staff. The trial enrolled residents and staff at 74 skilled nursing and assisted living facilities in the United States that had at least 1 confirmed case of SARS-CoV-2. Bamlanivimab reduced the incidence of COVID-19 among participants at high risk of severe disease while also lowering the rates of infection in resident and individuals at high risk. Deaths attributed to COVID-19 during the course of the trial occurred only among the participants of the placebo group. However, no significant difference was found regarding the incidence of COVID-19 or SARS-CoV-2 infection in low-risk staff participants in the bamlanivimab arm compared with the placebo arm [56]. These findings are in accordance to the hypothesis that mAbs administration is especially effective in older and high risk individuals.

Part A of the Regeneron trial (NCT04452318) consists of a randomized, double-blind, placebo- controlled trial to assess the efficacy and safety of the subcutaneous administration of casirivimab plus imdevimab (600/600 mg) in preventing SARS-CoV-2 infection among previously uninfected household contacts of infected persons without previous or ongoing infection. Symptomatic SARS-CoV-2 infection developed in 1.5% of participants in the casirivimab/imdevimab arm and in 7.8% in the placebo arm (relative risk reduction, 81.4%; odds ratio, 0.17; *p* < 0.001) [57].

In September 2021, and following the results of these trials, the FDA issued EUAs to allow the emergency use of bamlanivimab/etesevimab (iv) and casirivimab/imdevimab (iv or subcutaneous) for post-exposure prophylaxis of COVID-19 in individuals at high risk of progression to severe COVID-19, hospitalization or death, (patients who have not yet completed a full vaccination course or who are not expected to develop an appropriate immune response vaccination, i.e., immunocompromised individuals) who have been exposed to SARS-CoV-2 infection (close contact with an individual infected with SARS-CoV-2) or who are at high risk of exposure to an individual infected with SARS-CoV-2 because of occurrence of SARS-CoV-2 infection in other individuals in the same institutional setting (e.g., nursing homes, prisons) [36,38,58].

Preliminary and interesting results in preventing SARS-CoV-2 infection in a rhesus macaque model are associated to the combination of two long-acting antibodies (cilgavimab + tixagevimab), developed from the B-cells of a convalescent donor after the infection [40,42]. These mAb have been engineered to have a longer half-life, to provide an expected 6–12 months of protection after a single intramuscular administration. Two clinical trials are ongoing in both pre- and post-exposure prophylaxis setting (PROVENT trial (NCT04625725); STORM CHASER trial (NCT04625972)) [59,60]. The PROVENT trial was conducted among participants who would supposedly benefit from prevention with long-acting antibodies due to an increased risk of inadequate response to active immunization or an increased risk of SARS-COV 2 infection, including those whose locations or circumstances put them at appreciable risk of exposure to the SARS-CoV [60]. It is likely that this population will be the most eligible for the use of long-acting mAbs.

### 3.2. Use of mAb in Patient Hospitalized for COVID-19

Data supporting the use of casirivimab 4000 mg plus imdevimab 4000 mg in hospitalized patients with COVID-19 and with a negative serology for the anti-S protein antibody come from the RECOVERY study. No difference was found in 28-day all-cause mortality between standard of care and standard of care with intravenous casirivimab/imdevimab. However, in the subgroup of patients seronegative for the anti-S protein antibody, a significant reduction in 28-day all-cause mortality was found in the casirivimab plus imdevimab arm: 396 of 1633 patients (24%) died in the casirivimab plus imdevimab arm compared to 451 of 1520 patients (30%) in the standard of care arm (rate ratio 0.80; 95% CI, 0.70–0.91; *p* = 0.001) [61].

## 4. Challenges for Using mAbs against SARS-CoV-2

### 4.1. Activity against SARS-CoV-2 Variants

Viruses, including SARS-CoV-2, change over time. Most changes have a negligible impact; however, some may affect several characteristics of the virus, such as how easily it spreads, the associated disease severity, or the performance of the existing vaccines against it. Mutations in the SARS-CoV-2 spike protein could affect mAbs efficacy. Different phylogenetic nomenclatures have been used for the currently circulating SARS-CoV-2 variants. The S protein has different hotspots of mutation and deletion: those eminently involved in the immune escape process are within the RBD, such as K417N/T, N439K, L452R, Y453F, S477N, E484D/K/Q, and N501Y. It is not clear how frequently these mutations occur in mAbs-treated-patients or how they influence virus clearance.

Convergent evolution has led to different combinations of mutations among different variants. Therefore, monitoring mutations is necessary to forecast and re-adapt the inventory of therapeutic solutions [62].

The World Health Organization (WHO) has been monitoring and assessing the evolution of SARS-CoV-2 since January 2020. Although the mutation rate of SARS-CoV-2 is lower than other RNA viruses such as the influenza virus, probably due to the viral internal proofreading mechanism, a timely detection of these mutations and their impact on treatment efficacy, or pandemic countermeasures, are very important.

The established nomenclature systems for naming and tracking SARS-CoV-2 genetic lineages by GISAID, Nextstrain, and Pango are currently and will remain in use by scientists and in scientific research. At the present time, WHO experts recommend using letters of the Greek Alphabet, i.e., Alpha, Beta, Gamma, Delta, which will be easier and more practical to be discussed by non-scientific audiences. SARS-CoV-2 variants associated with “clinical evidence” of greater transmissibility, altered virulence, or the ability to escape natural infection- and vaccine-mediated immunity or current diagnostic tests are called Variants of Concern (VoC). Other variants that posed a “potentially increased risk” to global public health are called Variants of Interest (VoI). During late 2020, the emergence of variants posing a threat to global public health prompted the characterization of specific VoCs and VoIs. See Table 3 and Table 4 for the currently designated VoC and VoI [63].

Monitoring resistance to mAbs among the new variants will be key to defining whether some of the newly developed mAbs should be discontinued or if different combinations should be investigated [64].

Hereafter, we report the susceptibility of the currently designated VoC to the mAb in clinical use:

- *Alpha (B.1.1.7) variant:* This VoC maintains in vitro susceptibility to all the mAbs against SARS-CoV-2 that are currently approved through EUAs [36,38].
- *Beta (B.1.351) variant:* This VoC includes the E484K and K417N mutations, which results in a reduction in in vitro susceptibility to bamlanivimab and etesevimab [36,65]. In vitro studies also suggest that this variant has markedly reduced susceptibility to casirivimab, although the combination of casirivimab and imdevimab appears to retain activity; sotrovimab appears active as well against this VoC [38,39].
- *Gamma (P.1) variant:* This VoC includes the E484K and K417T mutations, which results in a marked reduction in in vitro susceptibility to bamlanivimab and etesevimab [36,66]. Additionally, this variant shows reduced susceptibility to casirivimab, although the combination of casirivimab and imdevimab appears to retain activity; sotrovimab appears to retain activity as well [38,39].
- *Delta (B.1.617.2) variant:* This is the prevalent VoC in the United States. It contains the L452R mutation, which results in a modest decrease in in vitro susceptibility to the combination of bamlanivimab and etesevimab, although the clinical implications of this finding are not fully known. Sotrovimab and casirivimab plus imdevimab appear to maintain activity [38,39,67].

A recent study by Vellas et al. evaluated the impact of neutralizing mAbs therapy on the nasopharyngeal viral load and the emergence of SARS-CoV-2 variants. Thirty-two SARS-CoV-2 infected patients were treated with neutralizing mAbs, 17/32 were immunocompromised (including 11 solid organ transplant patients). Most patients (31.97%) harbored the B.1.1.7 variant; one had the B.1, clade 20A. Four patients were given Bamlanivimab, 23 were given Bamlanivimab/ Etesevimab, and 5 were given Casirivimab/ Imdevimab. The analysis of the SARS-CoV-2 S protein evolution in infected patients treated with mAbs indicated that key mAb activity-reducing mutations (Q493R/K, 139 E484K) appeared in 5 immunocompromised patients treated with Bamlanivimab/Etesevimab, suggesting that immunosuppression could impair virus elimination, enhancing the risk of viral mutation under mAbs. No new key mutations in patients treated with Casirivimab/Imdevimab patients were found [68].

Mutations of SARS-CoV-2 Spike protein in VoC and resistance profile of clinical mAbs are summarized in Table 5.

### 4.2. Innovative Way of mAbs Administration

Another major mAbs hurdle, in both ambulatory outpatient and prophylaxis settings, is represented by the route of administration. MAbs currently approved by the FDA for treatment of COVID-19 are administered by intravenous infusion. Subcutaneous injection is a possible alternative for the combination casirivimab plus imdevimab exclusively, when intravenous infusion is not feasible and would lead to delay in treatment [38]. Part B of the Regeneron trial (NCT04452318) evaluated the efficacy and safety of subcutaneous casirivimab and imdevimab combination to prevent progression from early asymptomatic SARS-CoV-2 infection to COVID-19. This combination significantly prevented progression from asymptomatic to symptomatic disease compared with placebo (31.5% relative risk reduction)). The authors concluded that subcutaneous administration of this mAb prevented progression from asymptomatic SARS-CoV-2 infection to COVID-19 and was well tolerated [57].

Specific infusion centers, pop-up sites, or in-home visits may be safer from a public health context while offering more convenient services to the patient [54]. Several studies are focusing on mAbs administration via nasal sprays or aerosolized formulations [70,71,72,73,74,75,76]. Early clinical studies have confirmed that this approach is safe and can be used to prevent and treat SARS-CoV-2 infection [77]. The preclinical study on COVID-19-inhaled mAb demonstrated their therapeutic efficacy in animal models (mice and hamsters), significantly reducing the viral burden in SARS-CoV-2-infected animals when used either prophylactically or therapeutically [70,78]. Regarding the ongoing clinical trials, researchers are studying foralumab (administered by nasal spray) to treat COVID-19. Foralumab is a fully human second generation anti-CD3 mAb with a modified Fc unit composed of two heavy chains with an immunoglobulin region and two light chains with a kappa constant region.

In a Brazilian study, it was observed that in 39 patients with mild to moderate COVID-19, intranasal foralumab may be of benefit in modulating immune reactivity and in reducing pulmonary inflammation [79]. Moreover, adeno-associated virus vectors may offer opportunities for delivering mAb-expressing gene therapy constructs [54,73].

## 5. Global Access

At the beginning of the SARS-CoV-2 pandemic, obtaining antibodies from patients with COVID-19 was considered a fruitful strategy to recover human specialized neutralizing antibodies. However, there has been little discussion on mAbs production technologies and how to obtain quality mAbs that are safe, efficient, and accessible to the global population [80,81]. An important limitation to the use of mAbs in clinical settings is their high cost [82]. Furthermore, a major barrier to global mAbs access is that many of these innovative products are not even available in many low- and middle-income countries. Advancements in antibody optimization, manufacturing technologies, and packaging and delivery have the potential to lower mAbs production costs and increase efficiency [83]. Therefore, identifying the patients who could receive the highest benefit from these mAbs shall become a priority, to maximize the appropriate use of resources [84].

## 6. Conclusions

So far, almost 200 million people have been fully vaccinated in the US, about 56% of the population [85]. Globally, more than 6 billion vaccine doses have been administered [86].

Now that vaccines are being widely deployed, the question is what could be the most appropriate role for monoclonal antibodies. MAbs should be reserved for individuals at high risk of developing a serious COVID-19 illness despite the SARS-Cov-2 vaccination, such as immunocompromised patients (primary or secondary immunodeficiencies). Additionally, mAbs could prove helpful for people who cannot become vaccinated due to medical contraindications or personal choice and those who remain at risk of severe SARS-CoV-2 infection due to certain medical conditions (e.g., obesity, diabetes, and COPD) with low level or absence of neutralizing antibodies.

SARS-COV-2 is constantly changing, and mutations in its surface spike proteins may render it unrecognizable to the immune system of the host. Therefore, it is crucial that the scientific research community perseveres in meticulously monitoring these changes and in developing treatments and vaccines to tackle troublesome variants of concern.

## 7. Research Strategy and Selection Criteria

References for this review were identified from PubMed, Embase, and Cochrane with the research terms: “monoclonal antibodies”, “SARS-CoV-2”, “COVID-19”, “prophylaxis”, “therapy” and “variants” with several combinations. Only papers in English were included. The final reference list was generated based on timeline, originality, and relevance to the scope of this review.

## Figures and Tables

**Table 1:** Risk factors for progression to severe COVID-19 [47].

Age ≥ 65 years
Obesity or being overweight (e.g., adults with BMI > 25 kg/m^2^, or if age 12–17, have BMI ≥85th percentile for their age and gender based on CDC growth charts)
Pregnancy
Chronic kidney diseases
Diabetes
Immunosuppressive diseases or immunosuppressive treatment
Cardiovascular disease (including congenital heart disease) or hypertension
Chronic lung diseases (e.g., chronic obstructive pulmonary disease, asthma, interstitial lung disease, cystic fibrosis and pulmonary hypertension)
Sickle cell disease
Neurodevelopmental disorders (e.g., cerebral palsy) or other conditions that confer medical complexity (for example, genetic or metabolic syndromes and severe congenital anomalies)
Medical-related technological dependence (e.g., tracheostomy, gastrostomy, or positive pressure ventilation (not related to COVID-19))

**Table 2:** Randomized clinical trials supporting mAbs approved by FDA EUAs.

mAb	Study Design	Methods	Results
**Bamlanivimab plus etesevimab** (Trial Number NCT04427501)	Double-blind, phase 3 randomized clinical trial in outpatients with mild to moderate COVID-19 who are at high risk for progressing to severe COVID-19 and/or hospitalization [48].	**Intervention:** Single intravenous infusion of: - bamlanivimab 2800 mg + etesevimab 2800 mg - Placebo **Primary endpoint:** Proportion of participants with COVID-19 related hospitalization or death by any cause by day 29	**Number of Participants:** - bamlanivimab + etesevimab (*n* = 518) - placebo (*n* = 517) **Primary outcome:** Proportion of participants with COVID-19 related hospitalization or death by any cause by Day 29: 11 of 518 participants (2.1%) in the bamlanivimab + etesevimab arm vs. 36 of 517 (7.0%) in the placebo arm (absolute risk difference, −4.8 percentage points; 95% confidence interval (CI), −7.4 to −2.3; relative risk difference, 70%; *p* < 0.001). Proportion of participants who had died from any cause by Day 29: 0 of 518 participants (0%) in the bamlanivimab + etesevimab arm vs. 10 of 517 (1.9%) in the placebo arm (*p*< 0.001).
**Casirivimab plus imdevimab** (Trial Number NCT04425629)	Double-blind, Phase 3 RCT in outpatients with mild to moderate COVID-19 [49,50].	**Intervention:** Single intravenous infusion of: - casirivimab 600 mg + imdevimab 600 mg - casirivimab 1200 mg + imdevimab 1200 mg - placebo **Endpoint:** Proportion of patients with COVID-19-related hospitalization or all-cause death through Day 29	**Number of participants:** - casirivimab 600 mg + imdevimab 600 mg (*n* = 736) vs. placebo (*n* = 748) - casirivimab 1200 mg + imdevimab 1200 mg (*n* = 1355) vs. placebo (*n* = 1341) **Primary outcomes:** COVID-19-related hospitalization or all-cause death through Day 29: - 7 of 736 (1.0%) in casirivimab 600 mg plus imdevimab 600 mg arm vs. 24 of 748 (3.2%) in placebo arm (relative risk reduction, 70.4%; 95% CI, 31.6 to 87.1; *p* = 0.0024) - 18 of 1355 (1.3%) in casirivimab 1200 mg plus imdevimab 1200 mg arm vs. 62 of 1341 (4.6%) in placebo arm (relative risk reduction 73%; CI 95%, 51.7 to 82.9; *p* < 0.001).
**Sotrovimab** (Trial number NCT04545060)	Double-blind, Phase 1/2/3 RCT in outpatients with mild to moderate COVID-19 [51].	**Interventions:** - sotrovimab 500 mg IV - Placebo **Primary Endpoint:** Proportion of patients with hospitalization or death from any cause by Day 29	**Number of Participants:** - sotrovimab (*n* = 291) - placebo (*n* = 292) **Primary outcome:** There an 85% relative risk reduction in all-cause hospitalizations or deaths in patients who received sotrovimab compared to those who received placebo. (All-cause hospitalization or death by Day 29: 3 of 291 (1%) in sotrovimab arm vs. 21 of 292 (7%) in placebo arm (*p* = 0.002))

**Table 3:** Currently designated Variants of Concern (VoC) [63].

WHO Label	Pango Lineage *	GISAID Clade	Nextstrain Clade	Additional Aamino Acid Changes Monitored °	Earliest Documented Samples	Date of Designation
Alpha	B.1.1.7 ^#^	GRY	20I (V1)	+S:484K +S:452R	United Kingdom, Sep-2020	18 December 2020
Beta	B.1.351	GH/501Y.V2	20H (V2)	+S:L18F	South Africa, May-2020	18 December 2020
Gamma	P.1	GR/501Y.V3	20J (V3)	+S:681H	Brazil, Nov-2020	11 January 2021
Delta	B.1.617.2 ^§^	G/478K.V1	21A, 21I, 21J	+S:417N	India, Oct-2020	VOI: 4 April 2021 VOC: 11 May 2021

+ Relative changes in global prevalence of VOCs can be visualized in the COVID-19 Weekly Epidemiological Updates, available here: https://www.who.int/emergencies/diseases/novel-coronavirus-2019/situation-reports (accessed on 15 November 2021). * Includes all descendent lineages. The full list of Pango lineages can be found here: https://cov-lineages.org/lineage_list.html (accessed on 15 November 2021); for FAQ, visit: https://www.pango.network/faqs/ (accessed on 15 November 2021). ° only found in a subset of sequences. ^#^ includes all Q* lineages (in the Pango nomenclature system, Q is an alias for B.1.1.7). ^§^ includes all AY* lineages (in the Pango nomenclature system, AY is an alias for B.1.617.2).

**Table 4:** Currently designated Variants of Interest (VoI) [63].

WHO Label ^§^	Pango Lineage *	GISAID Clade	Nextstrain Clade	Earliest Documented Samples	Date of Designation
Lambda	C.37	GR/452Q.V1	21G	Peru, December 2020	14 June 2021
Mu	B.1.621	GH	21H	Colombia, January 2021	30 August 2021

* includes all descendent lineages. The full list of Pango lineages can be found here: https://cov-lineages.org/lineage_list.html; for FAQ, visit: https://www.pango.network/faqs/ (accessed on 15 November 2021). ^§^ Former VOIs currently designated as VUMs: Kappa: B.1.617.1; Iota: B.1.526; Eta: B.1.525; Epsilon: B.1.427/B.1.429. Former VOIs no longer designated as VUMs: Zeta: P.2; Theta: P.3.

**Table 5:** Mutations of SARS-CoV-2 S in VOC and resistance profile of clinical mAbs [69].

	Casirivimab Indevimab	Bamlanivimab Etesevimab	Sotrovimab	Cilgavimab Tixagevimab	Regdanvimab
**B.1.1.7 (UK)**	S S	S S	S	S S	S
**B.1.351 (South Africa)**	R S	R R	S	S S	I/R
**P.1 (Brazil)**	R S	R R	S	S S	Pot I/R
**B.1.429 (California)**	S S	R S	S	S S	I/R
**B.1.1.258 (Scotland)**	S R	S U	S	U U	Pot S
**B.1.525 (Nigeria)**	Pot I/R Pot S	Pot I/R Pot S	S	Pot S Pot S	U
**B.1.526 (New York)**	Pot I/R Pot S	Pot I/R Pot S	S	Pot S Pot S	U
**B.1.617.1 (India)**	S S	R S	S	Pot S Pot S	U

S = neutralized (<10-fold loss of neutralization). I/R = poorly or not-neutralized (>10-fold loss of neutralization). Pot S = potential S. Pot I/R = potential I/R. U = Unknown.

## Data Availability

Data sharing not applicable.
